# Accurate rare variant phasing of whole-genome and whole-exome sequencing data in the UK Biobank

**DOI:** 10.1038/s41588-023-01415-w

**Published:** 2023-06-29

**Authors:** Robin J. Hofmeister, Diogo M. Ribeiro, Simone Rubinacci, Olivier Delaneau

**Affiliations:** Department of Computational Biology, University of Lausanne, Lausanne, Switzerland

**Keywords:** Population genetics, Software

## Abstract

Phasing involves distinguishing the two parentally inherited copies of each chromosome into haplotypes. Here, we introduce SHAPEIT5, a new phasing method that quickly and accurately processes large sequencing datasets and applied it to UK Biobank (UKB) whole-genome and whole-exome sequencing data. We demonstrate that SHAPEIT5 phases rare variants with low switch error rates of below 5% for variants present in just 1 sample out of 100,000. Furthermore, we outline a method for phasing singletons, which, although less precise, constitutes an important step towards future developments. We then demonstrate that the use of UKB as a reference panel improves the accuracy of genotype imputation, which is even more pronounced when phased with SHAPEIT5 compared with other methods. Finally, we screen the UKB data for loss-of-function compound heterozygous events and identify 549 genes where both gene copies are knocked out. These genes complement current knowledge of gene essentiality in the human genome.

## Abstract (web summary)

SHAPEIT5, a phasing method that accurately processes large sequencing datasets, was applied on the UK Biobank whole-genome and whole-exome sequencing data to generate reference panels of haplotypes that boost imputation accuracy and enable the detection of compound heterozygous loss-of-function events for 549 genes.

## Main

Modern genetic association studies are increasingly based on whole-genome or whole-exome sequencing (WGS/WES) for hundreds of thousands of samples collected as part of nationwide biobanking initiatives^1,2^. Compared with previous studies based on single nucleotide polymorphism (SNP) arrays, WGS and WES data can identify rare variants (e.g., minor allele frequency below 1%), allowing a systematic characterization of their contribution to trait heritability^3^, functional relevance^4^ and effects on various traits and diseases^5,6^. In this context, haplotype phasing of rare variants, which involves distinguishing the two parentally inherited copies of each chromosome into haplotypes, adds a layer of biologically relevant information and unlocks new analyses. For instance, phasing is crucial to identify compound heterozygous events, which occur when both copies of a gene contain nonidentical, heterozygous mutations. In the case of Mendelian disorders, compound heterozygosity is one of the most common inheritance models for rare recessive diseases in nonconsanguineous individuals^7,8^. Previous efforts to identify compound heterozygous events in large cohorts provided valuable insights, yet these either relied on imputed data^9^ or ignored phasing information^6^. Compound heterozygous event identification requires high-confidence phase information to be considered when rare variants are analyzed, such as in gene-based burden test analysis^10^. The most common approach to phase rare variants without parental genomes or long-reads in large cohorts of individuals is statistical phasing, which leverages information across individuals to make estimation of haplotypes^11^. This technique is well established for common variants typed on SNP arrays, where phase information is used, for instance, to perform genotype imputation^12^, admixture analysis^13^ and genealogy estimation^14^. Phasing methods have been optimized to scale to the thousands of samples in modern SNP array datasets, and the time is ripe to do the same for the millions of rare variant sites present in WGS/WES datasets. As an example, the WGS data for 150,119 UKB samples comprise three orders of magnitude more variants than the Axiom array data, around 96% of them having a minor allele frequency (MAF) below 0.1%. Phasing large scale WGS/WES datasets is challenging and new methods able to handle large amounts of rare variants are now emerging^15^. Recently, a computationally efficient solution for rare variant phasing has been implemented in Beagle v.5.4 (refs. ^16,17^), in which common and rare variants are phased separately: in a first step, a standard phasing method is used to obtain haplotypes at common variants, and in a second step rare heterozygous sites are phased onto the resulting haplotypes using genotype imputation technique. This type of strategy, based on haplotype scaffolds, has been used in other contexts, such as in genotype imputation^18^, integration of family data^19^ and external phasing information^20^.

In this work, we describe SHAPEIT5, a method designed to accurately phase rare variants in large WGS/WES datasets, including singletons, with moderate accuracy, while attributing phasing confidence scores. We applied it to estimate haplotypes for 150,119 and 452,644 UKB samples with WGS and WES data, respectively. We demonstrate the benefit of using these two haplotype collections as reference panels for SNP array imputation and finally show that the phase inferred at rare variants in the WES dataset can be screened to reliably identify compound heterozygous loss-of-function (LoF) mutations, probably leading to complete gene knockouts.

## Results

### Overview of the SHAPEIT5 phasing method

**Fig. 1:**
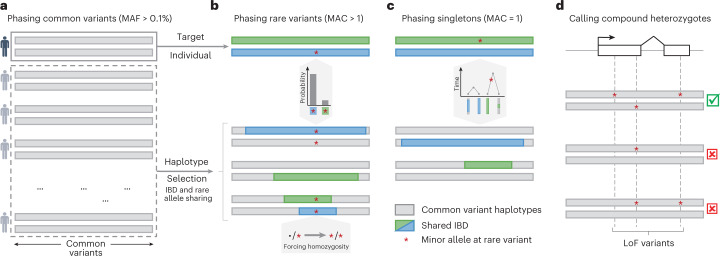
Rationale of SHAPEIT5. **a**, All samples are phased at common variants (MAF ≥ 0.1%). **b**, Phasing of a given rare variant onto the haplotypes at common variants. Conditioning haplotypes used in the estimation share long matches with the target (green and blue) and are not monomorphic at the rare variant. Since heterozygous genotypes for the rare variant are unphased, the minor alleles at those are assumed to be on both haplotypes (i.e., forcing homozygosity). **c**, Singleton phasing by assigning the new allele on the target haplotype with the shortest match. **d**, Compound heterozygous event mapping based on the rare variant phasing (**a**–**c**).

SHAPEIT5 performs haplotype phasing of WGS or WES data using three different phasing models, each focusing on a specific type of variants: (1) common variants are phased using the SHAPEIT4 model^20^, (2) rare variants are phased onto the resulting haplotypes using an imputation model and (3) singletons are phased using a coalescent-inspired model. See Fig. 1 for an illustration of the phasing scheme. Common variants are defined as having a MAF above 0.1% and are phased using an optimized version of the SHAPEIT4 algorithm, known to perform well on large sample sizes (Fig. 1a).

The resulting haplotypes are used in a second stage as a scaffold onto which rare variants (MAF < 0.1%) are phased one after another, following a methodology similar to that of Beagle v.5.4 (refs. ^16,17^). To cope with the large numbers of rare variants, SHAPEIT5 uses a sparse data representation for rare variants: only genotypes carrying at least one copy of the minor allele are stored in memory and considered for computation, thereby discarding all genotypes being homozygous for the major allele^21,22^. SHAPEIT5 phases each rare heterozygous genotype conditioning on a small number of informative haplotypes (Fig. 1b). For a specific rare variant, these conditioning haplotypes are chosen so that (1) they belong to samples being locally identical-by-descent (IBD) with the target sample and (2) they are polymorphic at the rare variant (that is, at least a few carry a copy of the minor allele). To comply with the first requirement, SHAPEIT5 uses a positional Burrows-Wheeler transform (PBWT) data structure^23^ built on all the scaffold haplotypes at common variants. This allows rapid identification of shared segments between haplotypes. To ensure representation of the minor allele in the conditioning set (second requirement), the method performs a second PBWT pass restricted to the subset of samples carrying a copy of the minor allele. This second pass is performed efficiently by leveraging the sparse representation of the genotypes. We then determine the alleles carried by the conditioning haplotypes at the rare variant of interest, which is straightforward when homozygous. However, when a conditioning sample is heterozygous, the allele carried by each of its two haplotypes is unknown. In this case, our model assumes that both haplotypes carry the minor allele as done in Beagle v.5.4 (refs. ^16,17^). Once the conditioning set of haplotypes is assembled, SHAPEIT5 uses the Li and Stephens model^24^ to get the most likely phase configuration of the rare allele by imputation (that is, either on its first or second target haplotype; Supplementary Fig. 1). The strength of our model resides in the guarantee that each rare heterozygous genotype is phased from a conditioning set containing long haplotype matches and carrying copies of the two possible alleles.

For singleton variants (minor allele count (MAC) of 1), SHAPEIT5 uses another phasing model that (1) assumes singletons to be recent mutation events and (2) leverages IBD sharing patterns between haplotypes to make inference (Fig. 1c). Specifically, our model identifies the longest possible match in the dataset for each target haplotype. By definition, these matches point to haplotypes sharing recent common ancestors with the target and their lengths indicate the number of generations separating them: the shorter the match, the older the common ancestor. Our model assumes that an older common ancestor means more time for a mutation to occur on that lineage and therefore assigns the minor alleles of singletons to the target haplotype with the shortest match^25^.

### Phasing UKB exomes and genomes

We used SHAPEIT5 to phase haplotypes for three different UKB sequencing datasets: (1) WGS data on chromosome 20 for 147,754 samples and around 13.8 million SNPs and indels after quality control, (2) WES data for 452,644 samples and around 26 million variants and (3) WGS data for the full set of 150,119 samples and around 603 million variants. For (1) and (2), we included only samples for which Axiom array data are available and excluded parental genomes for duos (parent–offspring pairs) and trios (parent–offspring triplets) to measure phasing accuracy in the offspring. Numbers of samples, trios, duos and variants after quality control are given in Supplementary Table 1. Phasing of the WES dataset was performed for each chromosome independently and phasing of the WGS was done in overlapping chunks of around 4.5 Mb on average to leverage parallelization on the UKB Research Analysis Platform (RAP). We compare the performance of our method with Beagle v.5.4 (refs. ^16,17^) (default parameters) on the WES and WGS datasets on chromosome 20.

### Phasing performance in the UKB data

**Extended Data Fig. 1:**
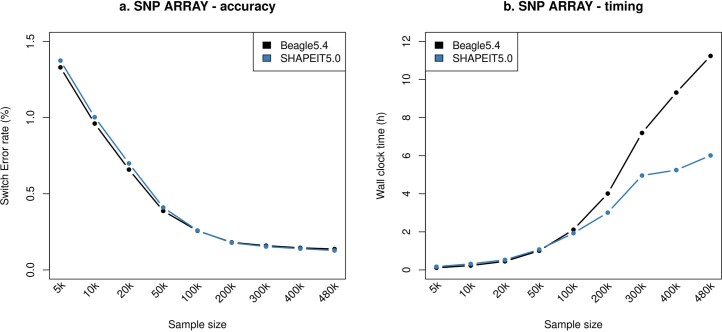
Switch error rates and running times on the UK Biobank Axiom array data. (**a**) SER computed at all variants typed on the UK Biobank Axiom array in duos and trios. (**b**) Phasing running times on the UK Biobank Axiom array. SER and running times have been computed for multiple downsampling experiments comprising 5000, 10000, 20000, 50000, 100000, 200000, 300000, 400000, 480000 samples (x-axis). SHAPEIT5 and Beagle5.4 are shown in blue and black, respectively. Source data

**Extended Data Fig. 2:**
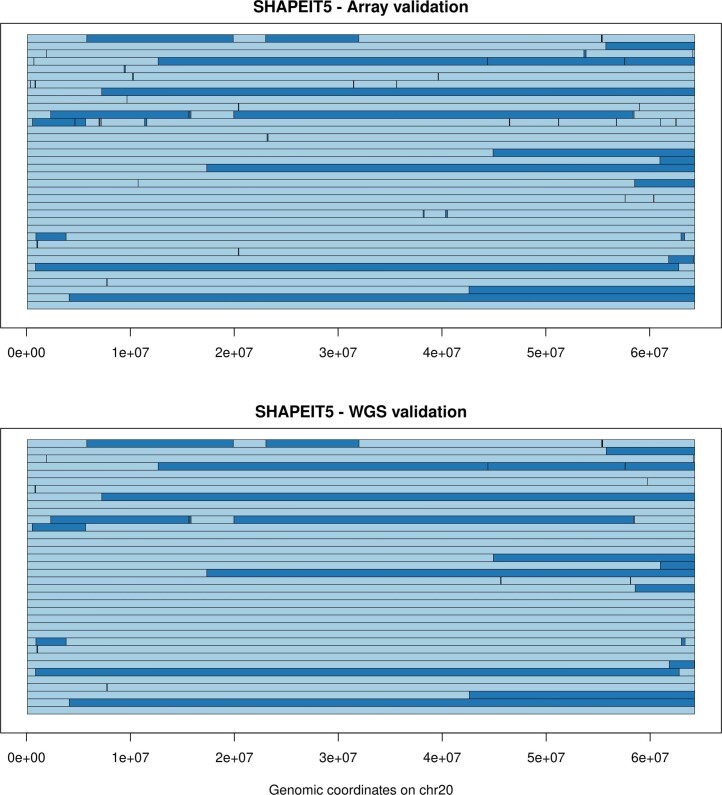
Chromosome 20 switch error locations. Switch error locations for 50 validation samples when phased as part of 480 k samples across the entire chromosome 20 (x-axis) with SHAPEIT5. A switch between dark and light blue represents a switch error. At the top is shown the Axiom array phasing when using Axiom array genotypes as validation and at the bottom when using the WGS data as validation genotypes. Many of the small segments in the top panel are due to genotyping errors in the duo/trio parents, which involve incorrect duo/trio phasing as validation. When using the WGS as validation, many of these inconsistencies disappear. Source data

To assess phasing performance, we used the available white British trios (719 for WES, 31 for WGS) and duos (432 for WGS). Using these, we (1) derived a true set of haplotypes for the offspring using inheritance logic, (2) performed statistical phasing of the WES and WGS datasets after having excluded parental genomes and (3) compared the offspring haplotypes obtained by statistical phasing with the true set obtained in (1). We assessed how close the two sets of haplotypes are by measuring the switch error rate (SER), which is the fraction of successive heterozygous genotypes phased differently. When looking at overall SER using different validation sets (duos, trios), different sets of variants (all variants or common variants only) and different sample sizes, we found minor differences between SHAPEIT5 and Beagle v.5.4 on the WGS data (Supplementary Fig. 2a–c). However, when considering only Axiom array positions, lower SER is observed with SHAPEIT5 (Supplementary Fig. 2d). We did not find the same pattern when phasing the Axiom array data only (*n* = 5,000 to *n* = 480,000): the two methods exhibit similar accuracy regardless of sample size (Extended Data Fig. 1). We obtained low SER (<0.2%) on the largest sample sizes for both methods, to the point that switch errors and genotyping errors cannot be distinguished (Extended Data Fig. 2).

**Fig. 2:**
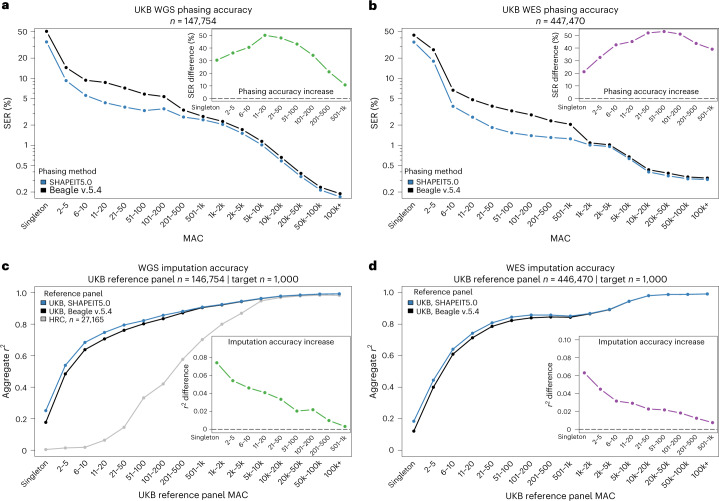
Phasing performance. **a**,**b**, SER (*y* axis, log scale) of SHAPEIT5 (blue) compared with Beagle v.5.4 (black) stratified by MAC (*x* axis) for the UKB WGS (**a**) and WES (**b**). The zoomed-in views show the relative reduction of SER using SHAPEIT5 compared with Beagle v.5.4 at rare variants. **c**,**d**, Imputation accuracy (Aggregate r2, *y* axis) for 1,000 white British samples genotyped with the Axiom array when using reference panels phased with either SHAPEIT5 (blue) or Beagle v.5.4 (black) WGS (**c**) or WES (**d**). In (**c**) the data were also imputed using the HRC reference panel (gray). The zoomed-in views show the increase of imputation accuracy at rare variants using the UKB dataset phased with SHAPEIT5 compared with Beagle v.5.4 as a reference panel. Source data

A key feature of the WES and WGS datasets is the large number of rare variants they contain. The number of heterozygous genotypes is low at these variants and they have a small contribution in global SER measurements. We therefore stratified the SER within bins of MACs to focus on rare variants. We assigned heterozygous genotypes to different MAC bins depending on the variant frequency and computed in each MAC bin the fraction of them being correctly phased (relative to the previous heterozygous genotype, regardless of its MAC). When doing so, we found that SHAPEIT5 phases rare variants with higher accuracy than Beagle v.5.4 in both the WGS and WES datasets (Fig. 2a,b). For instance, SHAPEIT5 and Beagle v.5.4 phase rare variants in the WGS data (MAC between 11 and 20) with SER of 4.36% and 8.76%, respectively, which is a 50.2% drop. In the WES dataset, the same variant category is phased by SHAPEIT5 with a switch error rate of 2.93% compared with 5.18% with Beagle v.5.4 (42.67% reduction). Overall, SHAPEIT5 phases rare variants in the WES and WGS with 20% to 50% fewer switch errors compared with Beagle v.5.4, depending on MAC. This improvement in accuracy is also observed when only using trios for validation (Supplementary Fig. 3) and depends on sample size (Supplementary Fig. 4). Significant differences between the two methods are observed in datasets comprising at least 50,000 samples and increase with sample size.

**Extended Data Fig. 3:**
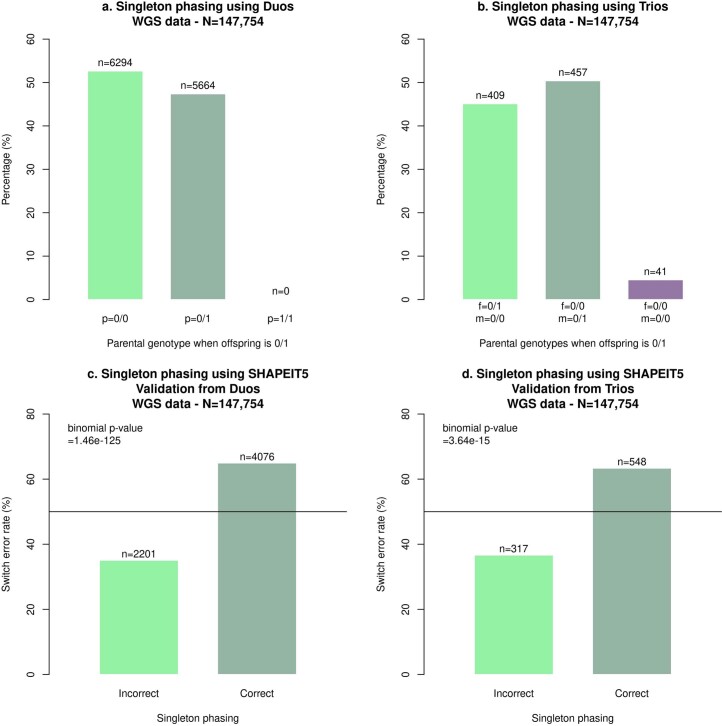
Singleton phasing. (**a**) Numbers of genotyped parents in duos being homozygous for the major allele (0/0) or heterozygous (0/1) for offspring being heterozygous at the singleton (0/1). Of note, a given variant is assumed to be a singleton here once the genotyped parent is excluded from the dataset to phase. (**b**) Numbers of parents in each genotype class for trios. (**c-d**) Numbers of singletons being incorrectly and correctly phased using duos (**c**) or trios (**d**) as validation. Binomial p-values were computed using the two sided *Exact binomial test* as implemented in R. Source data

In a large sequencing dataset, a singleton can be the product of several causes, including recent mutation, de novo mutation, somatic mutation or genotyping error. SHAPEIT5 aims to resolve the phase of recent mutations. We estimated the fraction of singletons falling in this category using duos and trios in the WGS data. We measured the fraction of singletons in offspring that is not supported by the genotype data available for the parents. In duos, we found that 47.36% of the singletons are supported by the genotyped parent, whereas 52.64% are not (Extended Data Fig. 3a), deviating from the expected 50% and suggesting that 5.26% of the singletons are not inherited from parents (assuming no inheritance bias). Consistently, in trios we found that 4.52% of the singletons in the offspring are not inherited from the parents (none of the parents carry the minor allele; Mendel inconsistency; Extended Data Fig. 3b). Together, this shows that most singletons (~95%) are inherited and can therefore be phased using both inheritance logic in trios and duos and our model. In the WGS dataset, we obtained SER of 35.1% and 36.6%, respectively (Extended Data Fig. 3c,d). In the WES dataset, we obtained an SER of 35.2% (Fig. 2b). While relatively high, this is a significant deviation from the expected 50% from previous models (binomial test *P* values <3.7 × 10^–15^; Extended Data Fig. 3c,d).

All computations were performed on the UKB RAP. The RAP offers a choice of two priority levels for computations: ‘spot’ (lower cost) and ‘on demand’ (higher cost). Assuming that all computing is performed on demand, Beagle v.5.4 and SHAPEIT5 require £57.80 and £65.20 of computing costs (as of October 2022) to phase chromosome 20 WGS data (*n* = 147,754), which correspond to approximately £2,890 and £3,258 for the entire genome (Supplementary Table 2). However, these are conservative estimates, as SHAPEIT5 allows phasing of the data in chromosomal chunks (in parallel), therefore greatly reducing the need for using ‘on demand’ priority.

### SHAPEIT5 phasing improves genotype imputation accuracy

Several downstream analyses in disease and population genetics require haplotype-level data. One example is genotype imputation^26^, which uses WGS data as a reference panel to predict missing genotypes in SNP array data. As the accuracy of genotype imputation depends on the reference panel, we quantified phasing errors using genotype imputation, which has two main advantages. First, it provides a validation alternative to SER that is easy to partition by minor allele frequency. Second, it assesses the phasing quality across all samples, and not only on a small subset with parental genomes available. We imputed a subset of 1,000 UKB British samples with SNP array data available, together with WGS and WES as validation.

**Extended Data Fig. 4:**
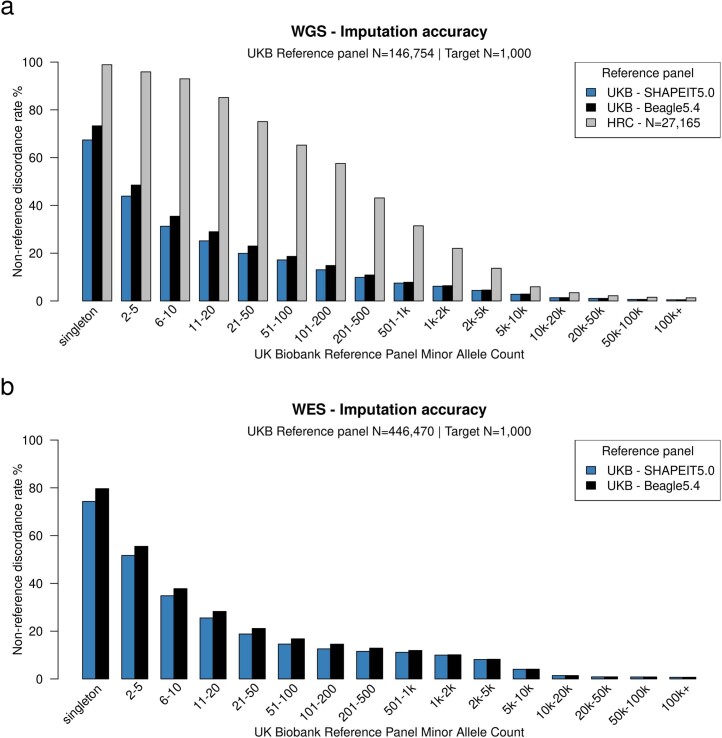
Non-reference discordance rate comparison between SHAPEIT5 and Beagle5.4. Non-reference discordance rate (NRD, y-axis) stratified by minor allele count (x-axis) for 1,000 white British samples genotyped with the Axiom array when using reference panels phased with either SHAPEIT5 (blue) or Beagle5.4 (black) whole genome sequencing (**a**) or whole exome sequencing (**b**). In (**a**) the NRD is also reported for the imputation experiment using the HRC reference panel (gray). Source data

First, we show that genotype imputation using the UKB WGS reference panel greatly outperforms the previous generation of reference panels, such as the Haplotype Reference Consortium (HRC)^27^ (Fig. 2c), in line with previous findings showing that large WGS panels enhance imputation^2^. For both UKB WGS and WES, we find that the reference panels phased with SHAPEIT5 outperform those phased with Beagle v.5.4 at rare variants (MAC < 500; Fig. 2c,d and Extended Data Fig. 4), consistent with the SER estimates reported in Fig. 2a,b. As an example, imputation using the WGS or WES reference panel phased with SHAPEIT5 provides an increase of squared Pearson coefficient of around 0.05 for variants with a MAC between 2 and 5. In an association study, this corresponds to an increase of 5% in effective sample size when testing these variants for association, due only to better reference panel phasing^28^. Even singletons are better imputed using the SHAPEIT5 panel. Despite the low overall accuracy at these variants, which restricts their utility in downstream analyses, this confirms on a larger scale the validity of our singleton phasing.

**Extended Data Fig. 5:**
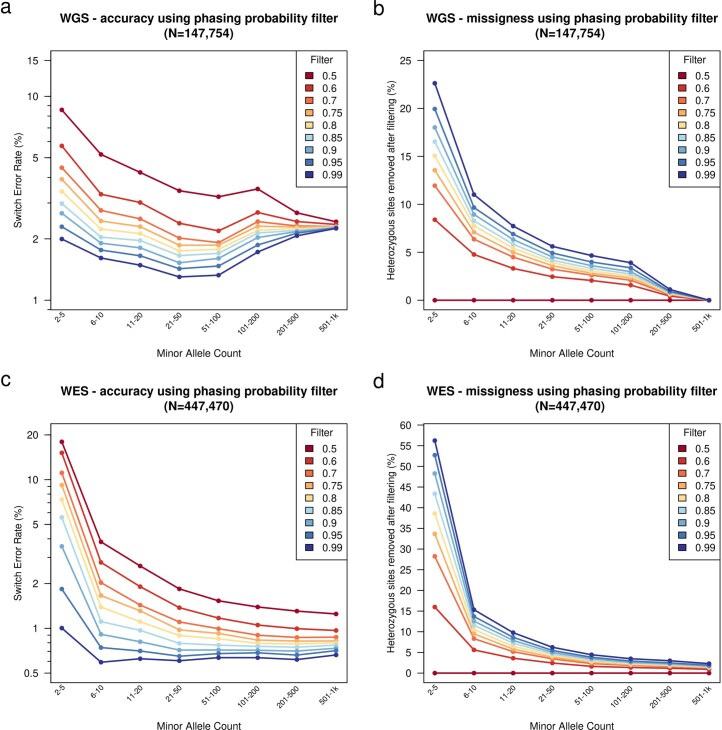
Performance of SHAPEIT5 phasing confidence score. Switch Error Rate (SER, y-axis) for WGS (**a**) and WES (**c**) datasets stratified by minor allele count (x-axis) at different phasing confidence score thresholds. Number of heterozygous sites filtered out at different phasing confidence score thresholds in the WGS (**b**) and WES (**d**) datasets. Filtering for a confidence score of 0.5 is equivalent to no filtering. Source data

SHAPEIT5 introduces a metric of phasing confidence at rare heterozygous genotypes (MAF < 0.1%), which corresponds to the probability of the reported phase. This allows controlling for phasing errors and utilizing phasing certainty in downstream analyses. Phasing confidence lies between 0.5 and 1, where 1 indicates no uncertainty in the phase and 0.5 means that the two phasing possibilities are equally likely. Singletons are attributed a phasing confidence of 0.5 as phasing confidence cannot be computed for them. We assessed the phasing accuracy at different confidence scores (Extended Data Fig. 5) and show that filtering variants with a threshold of 0.99 controls the SER to a maximum of around 2% for WGS data and around 1% for WES data while keeping most variants (for instance, >75% and >40% variants with MAC 2–5 are retained). This allows researchers to confidently use rare heterozygous genotypes in their analyses.

### Identification of LoF compound heterozygotes

**Fig. 3:**
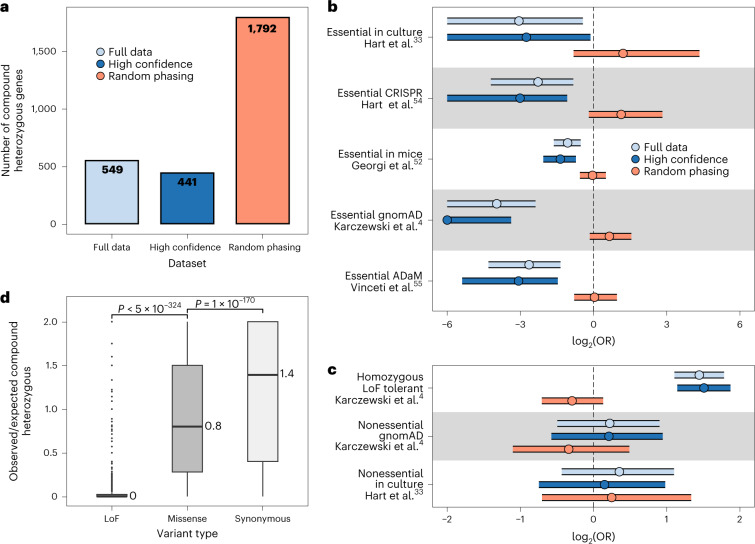
Compound heterozygous identification in the UKB WES data phased with SHAPEIT5. **a**, Number of genes with at least one individual with compound heterozygous LoF variants across several categories: Full data, all LoF variants in the study, except singletons; High confidence, LoF variants excluding calls with phasing confidence score <0.99; and Random phasing, shuffling phasing of all LoF variants (once). **b**, Two-way Fisher’s exact test odds ratios ± 95% confidence interval (log_2_-scaled) of compound heterozygous genes versus noncompound heterozygous genes presence in several lists of essential genes (Methods). Background is composed of 3,018 genes with ≥2 LoF mutations; *x* axis is capped at –6. **c**, Same as **b** but across lists of nonessential or LoF tolerant genes. **d**, Ratio between the number of individuals with compound heterozygous events and the expected number of individuals given the number of variants, per gene. Missense (*n* = 14,336 genes) and synonymous (*n* = 9,816 genes) events are shown in addition to LoF events (*n* = 2,150 genes) as a comparison. The length of the box corresponds to the interquartile range (IQR) with the center line and values corresponding to the median, and the upper and lower whiskers represent the largest or lowest value no further than 1.5× IQR from the third and first quartile, respectively. *P* values between categories correspond to two-sided Wilcoxon test *P* values. Source data

**Extended Data Fig. 6:**
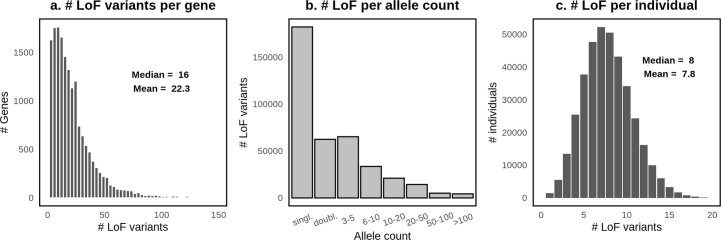
Frequency of loss-of-function (LoF) variants in the UK Biobank. (**a**) Number of LoF variants per protein-coding gene. 69 outlier values were excluded. (**b**) Number of LoF variants per allele count bin (N = 383,637). (**c**) Number of LoF variants per UK Biobank individual (N = 374,826). 11 outlier values were excluded. Source data

**Extended Data Fig. 7:**
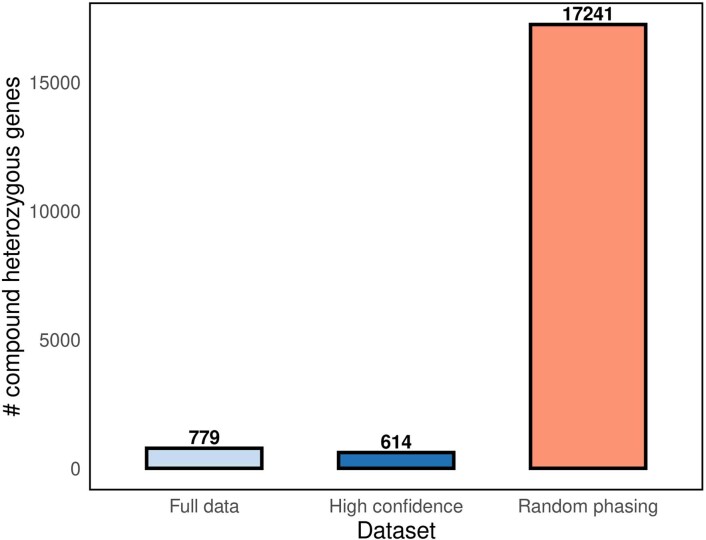
Number of compound heterozygous events across several categories. ‘Full data’: all LoF variants in the study, except singletons, ‘High confidence’: LoF variants excluding calls with phasing confidence score < 0.99, ‘Random phasing’: shuffling phasing of all LoF variants. Source data

Compound heterozygous events occur in an individual when both copies of a gene contain at least one heterozygous variant. Compound heterozygosity is often studied in the context of LoF variants, which are expected to have highly deleterious effects on genes—equivalent to a homozygous gene knockout. Indeed, compound heterozygous events have been linked to several diseases including cancer, birth defects and Alzheimer’s disease^8,29–32^. The accurate haplotype phasing across the UKB performed in this study, including extremely rare variants, allows the identification of individuals and genes with compound heterozygous events. For this, we gathered 383,637 high-confidence LoF variants (stop-gain, frameshift or essential splice variants) phased across 374,826 white British individuals and 17,689 protein-coding genes (Methods). We found that a gene has, on average, 22.3 LoF variants across the cohort and an individual has, on average, 7.8 LoF variants (Extended Data Fig. 6). To determine compound heterozygous events, we identify individuals with LoF mutations in both copies of a gene. Owing to their higher error rates and the risk of introducing false positives, we opted to exclude singletons from these analyses. A total of 2,150 (12%) out of 17,689 protein-coding genes tested had at least one individual with two or more LoF variants, and thus liable for compound heterozygous identification. From those 2,150 genes, we found 549 (26%) genes with one or more individuals with compound heterozygous LoF variants (Fig. 3a), for a total of 779 gene-individual events (766 distinct individuals; Extended Data Fig. 7 and Supplementary Data 1). When considering only high-confidence haplotype calls (phasing confidence score >0.99), we still identify 80% (441) genes and 79% (614) of the compound heterozygous events identified in the full dataset, indicating that these mostly rely on high-confidence haplotype calls (Fig. 3a and Extended Data Fig. 7). We found that the 549 compound heterozygous genes are highly depleted in several lists of known essential genes, compared with the 2,150 genes with two or more LoF variants (odds ratio (OR) 0.1–0.48 across essential gene lists, *P* < 9.7 × 10^−3^; Fig. 3b). Conversely, compound heterozygous genes are enriched in lists of nonessential and homozygous LoF tolerant genes (OR 1.2–2.7 across nonessential gene lists; Fig. 3c). The comparison with genes with two or more LoF variants in the same individual ensures that the signal observed is not due to the mere presence or absence of LoF variants in those genes, but rather the avoidance of them occurring in both gene copies. As the UKB is composed largely of healthy individuals, a depletion of compound heterozygous events in essential genes is expected.

**Extended Data Fig. 8:**
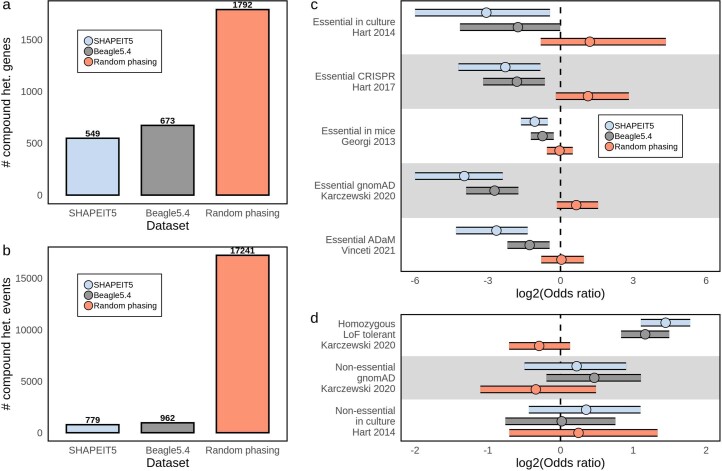
Compound heterozygous identification in the UK Biobank WES data phased with Beagle5.4. (**a**) Number of genes with a compound heterozygous event in at least one individual (N = 374,826 individuals). (**b**) Number of compound heterozygous events (N = 374,826 individuals). (**c**) Two-way Fisher’s Exact test odds ratios ± 95% confidence interval (log2-scaled) of compound heterozygous genes versus non-compound heterozygous genes presence in multiple lists of essential genes (see Methods). Background is composed of genes with ≥2 LoF mutations. X-axis is capped at −6. (**d**) Same as previous, but across lists of non-essential genes. Source data

When comparing with phasing performed with Beagle v.5.4, we found 673 compound heterozygous genes (962 events) that are significantly depleted in essential genes but at reduced levels compared with SHAPEIT5 phasing (Extended Data Fig. 8). Finally, as a control, we attributed the phase of variants randomly, which led to 1,792 compound heterozygous genes and 17,241 events (Fig. 3a), which did not display depletion in essential genes, as expected (Fig. 3b). Together, these results indicate that accurate haplotype phasing is crucial for the identification of bona fide compound heterozygous events.

**Extended Data Fig. 9:**
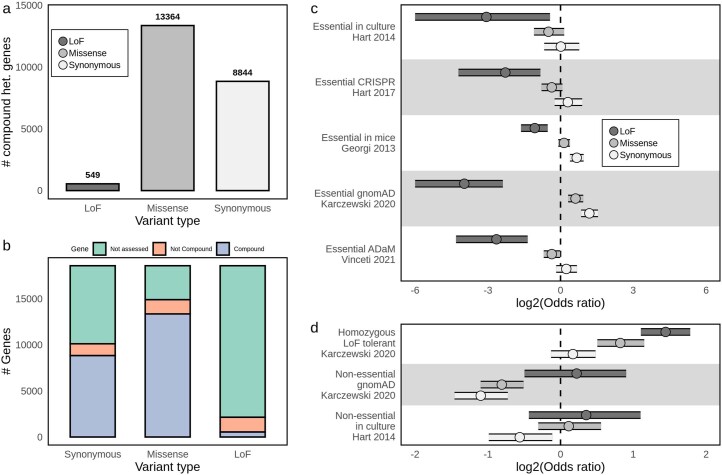
Comparison of compound heterozygous results with LoF, missense or synonymous variants. (**a**) Number of genes with a compound heterozygous event in at least one individual (N = 374,826 individuals). (**b**) From a total of 18,595 protein coding genes, number of genes with compound heterozygous events (blue), number of genes with individuals with ≥2 mutations but found only in one haplotype (orange), number of genes for which individuals with ≥2 mutations did not occur (green). (**c**) Two-way Fisher’s Exact test odds ratios ± 95% confidence interval (log2-scaled) of compound heterozygous genes versus non-compound heterozygous genes presence in multiple lists of essential genes (see Methods). Background is composed of 17,679 genes with ≥2 LoF mutations. X-axis is capped at −6. (**d**) Same as previous, but across lists of non-essential genes. Source data

The finding that compound heterozygous genes are depleted in essential genes indicates that such events are avoided, at least in a subset of the genes. To explore this further, we compared the number of expected and observed compound heterozygous events per gene, based on the variant distribution in the UKB cohort, assuming that each variant phase is independent (Methods). For LoF variants, we observed a marked decrease in observed compound heterozygous events compared with expected, confirming evidence for negative selection (Fig. 3d). Conversely, when considering variants with synonymous effect (Extended Data Fig. 9 and Supplementary Data 1), the number of observed compound heterozygous events is not depleted (median ratio = 1.4; Fig. 3d), indicating no or low selective pressure to reduce synonymous variant compound heterozygous events for most genes. When considering missense or low-confidence LoF variants (referred to as missense for simplicity), we observed a mild decrease in observed events compared with expected (mean ratio = 0.8; Fig. 3d and Supplementary Data 1), consistent with the possible deleterious effect of some missense variants. In addition, we found that missense compound heterozygous genes had only mild or no depletion for essential genes, whereas synonymous compound heterozygous genes either had no significant depletions or were even enriched in some essential gene sets (Extended Data Fig. 9). Overall, our results demonstrate that the accurate phasing at rare variants with SHAPEIT5 allows us to screen for compound heterozygous events across the UKB cohort with high confidence, revealing that LoF compound heterozygous events are under strong selective pressure in essential genes, as expected by their high negative impact.

## Discussion

We present SHAPEIT5, a tool for phasing rare variants in large sequencing datasets. SHAPEIT5 phases common variants first to create a haplotype scaffold. Subsequently, rare variants are phased one at a time on this scaffold. A key difference from Beagle v.5.4 is the use of individualized panels of haplotypes for rare variant phasing. SHAPEIT5 ensures representation of the minor alleles at rare variants, which leads to accuracy improvements that are more pronounced in larger sample sizes. We produced phased genomes for the UKB WGS and WES data for a compute cost below £4,000. The haplotype estimates have low SERs, with rare variants down to doubletons being phased with high confidence. This accurate phasing enables highly accurate genotype imputation when used as a reference panel. Beyond measuring error rates, we also validated phased haplotypes biologically by identifying compound heterozygous events, which we found highly depleted in essential genes, as expected. In addition, we achieved singleton phasing, albeit with higher error rates and therefore with limited downstream utility. However, we view this as an advance in phasing models as previous approaches were unable to phase singletons.

Although of substantial interest, previous knowledge of compound heterozygous cases comes mostly from case studies in families^7,8^ and there is currently no method to identify these events in large biobanks systematically. Here, we show that high-quality phasing of rare variants with SHAPEIT5 allows compound heterozygosity to be studied at the biobank-scale level, which can greatly increase the number of events characterized compared with the use of family data, in addition to exploring their association with new phenotypes. As a proof-of-principle, we screened all protein-coding genes for compound heterozygous events with high-confidence LoF variants and found 549 genes predicted to be fully knocked out across 816 UKB individuals out of the 374,826 individuals considered in this study. This complements other lists of nonessential genes^33^, with the main difference that these knockouts are found in vivo in humans. Approximately 0.22% of the UKB cohort had at least one gene knockout by compound LoF heterozygous events. This observed frequency of events matches previous estimates in outbred healthy cohorts^34^. UKB participants are not expected to have any rare and/or severe genetic diseases as their average age is 56 years, which is after the age of onset for most rare diseases. This partially explains why the gene knockouts observed are strongly depleted in several lists of essential genes. However, we still found 52 genes deemed as essential in at least one of the essential gene lists we analyzed. We can conceive three possible scenarios to explain these specific cases. First, the mutations had a moderate impact on the individual and did not result in severe disease. As an example, we found one individual with pulmonary embolism while having a knockout of the essential gene *ADAM19*—a gene reported for its involvement in pulmonary disease^35,36^. Second, compensatory mutations can rescue the deleterious effect of the knockout. For instance, we observed one individual with a knockout of *CFFTR*—an essential gene found to be rescued by several gain-of-function mutations across the genome^37–39^. Finally, some of the compound heterozygous events discovered may be false positives driven by incorrect phasing or erroneous LoF annotations.

We foresee that rare variant phasing in large sequencing studies such as the UKB has the potential to unlock many applications and analyses. First, other types of functional variants can be screened for compound heterozygous effects, for instance, combining LoF and missense or regulatory variants^40^. Second, phase information can be included in rare variant burden testing approaches, which usually consider only a mixture of the two haplotypes. Third, using accurately phased reference panels allows phasing of extremely rare variants with high accuracy, even singletons to some extent, for any new sequenced genome from the same population. This is beneficial for diagnosis of rare and severe diseases caused by compound heterozygous effects, such as in the Genomics England dataset^41^, in which diagnosis yield could be increased by incorporating phase information.

## Methods

### Ethics statement

This study relied on analyses of genetic data from the UKB cohort, which was collected with informed consent obtained from all participants. Data for this study were obtained under the UKB applications licence number 66995. All data used in this research are publicly available to registered researchers through the UKB data-access protocol.

### Common variant phasing

For common variant phasing (MAF ≥ 0.1%), SHAPEIT5 is based largely on the previous SHAPEIT version (v.4). Briefly, it updates the phase of each sample in turn within a Gibbs sampler iteration scheme: each sample is phased by conditioning on other samples’ haplotypes using the Li and Stephens model^24^. Two main features, already part of the SHAPEIT4 model, allow fast phasing at common variants: (1) first, the haplotype sampling step has linear complexity in the number of conditioning states^42^ and is multithreaded so that several samples are phased in parallel; (2) second, the sampling is based on a parsimonious and highly informative set of haplotypes, identified in constant time using the PBWT data structure.

However, one computational limitation of SHAPEIT4 resides in its inability to parallelize the construction of the PBWT, which can become relatively long in very large datasets. In SHAPEIT5, the main improvement we introduced for common variant phasing is a parallelization scheme for the PBWT construction: several PBWT passes are run in parallel on several central processing unit cores, each one running for a different chunk of 4 cM by default, achieving a notable reduction of the wallclock running time of the method.

### Rare variant phasing

To accurately phase rare variants (MAF<0.1%), SHAPEIT5 uses the haplotypes derived at common variants as haplotype scaffolds onto which heterozygous genotypes are phased one rare variant at a time. For a single heterozygous genotype, we aim to determine which of the two target chromosomes carries the minor allele (as opposed to the major allele). To do so, our method uses the Li and Stephens model to compute the probabilities of the two possible phases. The probabilistic inference is based on a set of haplotypes carried by other samples in the dataset, that we call conditioning haplotypes. Similarly, we call a conditioning sample, any sample carrying at least one conditioning haplotype and conditioning set, the collection of conditioning haplotypes used for inference. The outcome of the estimation is a posterior probability of the most likely phase for each of the rare heterozygotes. Specifically, our model comprises five main features:

#### Sparse representation

We use a sparse matrix representation of the genotypes at rare variants to efficiently store large amounts of genotype data in memory and speed up computations. Only genotypes carrying at least one copy of the rare allele are stored in memory together with the necessary indexes to determine the sample and variant to which the genotype corresponds. As most of the rare variants are homozygous for the major allele, this representation allows for a large reduction in memory usage and a fast identification of heterozygous genotypes at a given rare variant. To quickly retrieve rare genotypes at both the sample and variant levels, we store this sparse genotype matrix in memory together with its transpose.

#### Haplotype selection

To get the most informative haplotypes in the conditioning set, we require that they (1) share long haplotype matches with the target and (2) are not monomorphic at the rare variant of interest. The first condition ensures that the haplotypes in the conditioning set are informative for the copying model. The second condition ensures that the conditioning set contains carriers of the two possible alleles at the rare variant of interest. The latter is required to accurately contrast the two possible phasing possibilities of the rare heterozygous variant. To efficiently retrieve haplotypes complying with these properties, we use the PBWT data structure of the haplotype data derived at common variants. We perform both forward and backward PBWT sweeps so that we can identify long matches between haplotypes centered in the position of the rare variant by interrogating the flanking prefix arrays. This gives a first set of haplotypes that complies with condition (1), but not necessarily with condition (2). Therefore, we do a second identification of matches in the PBWT, this time restricting the search to the subset of samples carrying the minor allele. We achieve this second pass efficiently by taking advantage of the sparse genotype representation: we interrogate only the PBWT prefix arrays at the sparse indexes.

#### Forcing homozygosity

The conditioning set defined before contains a set of haplotypes that share large segments with the target haplotype at common variants, but they have not been phased yet at the rare variant of interest. When the conditioning sample (that is, the sample carrying the haplotype) is homozygous, this is not an issue as its two haplotypes carry the same allele. However, when the conditioning sample is heterozygous, we do not know the allele carried by each one of its two haplotypes. We solve this by simply assigning the minor allele to both haplotypes^17^. As a consequence of the two previous steps, the conditioning set of haplotypes is guaranteed to contain carriers of the two possible alleles at the rare variant of interest.

#### Copying model

We can now perform phasing of rare heterozygous genotypes based on the conditioning set of haplotypes that have been constructed as part of all the previous steps. SHAPEIT5 computes the probability that each target haplotype carries the minor allele by using a haploid version of the Li and Stephens model^24^ as implemented in Impute5 (ref. ^21^) (for a definition of the HMM parameters and a formal description of the imputation model used, see Rubinacci et al.^21^ and Howie et al.^43^). Specifically, it runs a forward-backward pass as done in the context of genotype imputation (see Marchini^44^ for details) to get the probabilities that each target haplotype carries the minor allele at the rare variant. In practice, the vector of copying probabilities is obtained at each rare variant by averaging the copying probabilities computed at the two closest flanking common variants. Here, the conditioning set of haplotypes serves as a local reference panel for imputing the alleles at the rare variant in the target sample. Of note, accurate inference is made possible since the conditioning set we chose is guaranteed to comprise carriers of both the major and minor alleles at the rare variant of interest. Having only carriers of a single allele would not be informative for making inference here. Finally, we use these imputation probabilities to derive phasing probabilities (Supplementary Fig. 1), which we can use to get the most probable phase or as phasing confidence scores to propagate phasing uncertainty in downstream analyses.

#### Singleton phasing

In the case of singletons, only the target sample carries a copy of the minor allele at the rare variant. Therefore, none of the conditioning haplotypes carries the minor allele and the whole copying model described above is unable to make inference. This is a well-known limitation of all statistical phasing methods. SHAPEIT5 can provide inference at these sites by using the Viterbi algorithm for the Li and Stephens model^24^, to obtain the longest shared IBD segment between each one of the two target haplotypes and the conditioning haplotypes. The minor allele of singletons is then assigned to the target haplotype with the shortest shared segment. The idea behind this model presumes that the shorter the IBD sharing between two haplotypes, the older their most recent common ancestor is, and therefore, the chance for new mutations to occur in that lineage is increased.

### Validation of haplotype estimates

To validate haplotype estimates, we use trios (two parents, one offspring) for WES data and both duos (parent–offspring pairs) and trios for WGS data. To identify parent–offspring relationships, we use the kinship estimate and the IBS0 as provided as part of the UKB SNP array release. We select parent–offspring relationships as having a kinship coefficient lower than 0.3553 and greater than 0.1767 and an IBS0 lower than 0.0012 (refs. ^1,45^). In addition, we require that the difference in age between parents and offspring is greater than 15 years and that the two parents have different sex for trios. We finally keep only self-declared white British individuals for which ancestry was confirmed by principal component analysis (PCA, UKB field 22006). The number of trios used in the validation for all three datasets (Array, WES or WGS) is shown in Supplementary Table 1. Validation of haplotypes is a two-step procedure. First, we statistically phase a given dataset including only the offspring samples. Second, we use the parents to measure the SER—a metric commonly used to assess how close estimated and true haplotypes are. The SER is defined as the fraction of successive pairs of heterozygous genotypes being correctly phased. In the context of this work, we measured SER stratified by bins of MAC. We assigned each heterozygous genotype to a given MAC bin and counted the fraction of heterozygous genotypes being correctly phased per MAC bin, relative to the previous heterozygous genotypes (this one can belong to any MAC bin). This definition of SER has the advantage of showing how well statistical phasing performs depending on the frequency of the variants it phases (either common or rare).

### UKB SNP array dataset

We used the UKB Axiom array in PLINK format and converted it into VCF format using plink2 (v.2.00a3.1LM). This resulted in 784,256 variant sites across autosomes for 488,377 individuals. We then applied quality control on the data using the UKB SNPs and samples QC file (UKB Resource 531) to only retain SNPs and individuals that have been used for the official phasing of the Axiom array data^1^, resulting in 670,741 variant sites across 486,442 individuals. This includes 897 white British parent–offspring trios and 4,373 white British parent–offspring duos (Supplementary Table 1).

### UKB WGS dataset

We use the whole-genome GraphTyper joint call pVCFs from the UKB RAP. We first decomposed multiallelic variants into biallelic variants using bcftools (v.1.15.1) norm -m^46^. We then performed quality control of the variant sites and filtered out SNPs and indels for (1) Hardy–Weinberg *P* value < 10^−30^, (2) more than 10% of the individuals having no data (GQ score = 0; missing data), (3) heterozygous excess less than 0.5 or greater than 1.5 and (4) alternative alleles with AAscore <0.5. Additionally, we kept only variant sites with the tag ‘FILTER = PASS’, as suggested by the data providers^15^. This resulted in a total of 603,925,301 variant sites, including 20,662,402 common variant sites (MAF ≥ 0.1%) and 583,262,899 rare variant sites (MAF < 0.1%), across a total of 150,119 individuals. This WGS dataset includes 31 trios and 432 duos (Supplementary Table 1). To assess the accuracy of the phasing, we use chromosome 20 only. For this analysis, we used only samples being also genotyped with the UKB Axiom array, resulting in 147,754 individuals (Supplementary Table 1). We phased chromosome 20 using chunks of, on average, 4.5 Mb with overlapping buffers of 250 kb. We used Beagle v.5.4 (refs.^16,17^) with default parameters on the entire chromosome 20.

### UKB WES dataset

We used the WES files in pVCF format as released on UKB RAP. The quality control pipeline has been described in Szustakowski et al.^47^. To phase WES data, we first merged it with the unphased SNP array data. The aim of this was to increase the number of common variants that are phased in the first step of SHAPEIT5 (that is, common variants phasing), which improves the quality of the haplotype scaffold onto which rare variants are phased, in particular at intergenic regions. We kept only individuals with both the SNP array and the WES data, resulting in 452,644 total individuals, including 719 white British parent–offspring trios and 3,014 white British parent–offspring duos. When a variant is listed in both the WES and the SNP array, we keep the SNP array copy as the SNP array is expected to be more robust to SNP calling errors^48^. This resulted in retaining a total of 26,199,614 variants, including 977,517 common variants (MAF ≥ 0.1%) and 25,222,097 rare variants (MAF < 0.1%) (Supplementary Table 1). Phasing the 452,644 individuals with both WES and Axiom array available data is performed for each chromosome independently in a single chunk. We also used Beagle v.5.4 (refs. ^16,17^) with default parameters.

### Genotype imputation

To perform genotype imputation from the phased WGS and WES datasets, we extracted 1,000 samples with British ancestry that are unrelated to any other sample in the dataset, and for which we had Axiom SNP array data available. We therefore used a reference panel composed of the remaining 146,754 WGS samples and 446,470 WES samples for both SHAPEIT5 and Beagle v.5.4. For the HRC reference panel, we used the PICARD toolkit (http://broadinstitute.github.io/picard/) to liftover the data to the Human genome assembly GRCh38, retaining 99.8% of the original variants.

We used Beagle v.5.4 for genotype imputation of SNP array data, allowing prephasing from the reference panel. We accessed imputation accuracy by measuring the squared Pearson correlation between imputed and high-coverage genotypes using the GLIMPSE_concordance tool^49^ (-gt-val option) at custom allele count bins (--ac-bins 1 5 10 20 50 100 200 500 1000 2000 5000 10000 20000 50000 100000 146754 for WGS, --ac-bins 1 5 10 20 50 100 200 500 1000 2000 5000 10000 20000 50000 100000 446470 for WES). A drop of correlation quantifies the reduction in effective sample size in association testing due to imperfect imputation. For instance, a difference of 0.05 involves a power loss equivalent to losing 5% of the data.

We also evaluated the nonreference discordance rate using the GLIMPSE_concordance^49^ tool. The nonreference discordance^46^ is calculated as NRD = (*e*_rr _+ *e*_ra _+ *e*_aa_)/(*e*_rr _+ *e*_ra _+ *e*_aa _+ *m*_ra _+ *m*_aa_), where *e*_rr_, *e*_ra_ and *e*_aa_ are the counts of the mismatches for the homozygous reference, heterozygous and homozygous alternative genotypes, respectively, and *m*_ra_ and *m*_aa_ are the counts of the matches at the heterozygous and homozygous alternative genotypes. NRD is an error rate that excludes the homozygous reference matches, which are the most frequent at rare variants, giving more weight to the other matches. We computed the nonreference discordance rate within frequency bins in the reference panel.

### Compound heterozygosity detection

We restricted the analysis to the cohort of self-declared white British individuals for which the ancestry is confirmed by PCA (UKB field 22006) with both SNP array and exome-seq data, excluding parental individuals (*n* = 374,826). Only WES variants with MAF < 0.1% before sample filtering were considered. Variant annotations (LoF, Synonymous and Missense|LC) were obtained from the Genebass database^50^ through Hail (gene-level results, results.mt). Briefly, these variants had been annotated by Ensembl VEP v.95 (ref. ^51^) and LoF variants (stop-gain, frameshift and splice donor/acceptor sites) were further processed by LOFTEE^4^, separating high-confidence (used as ‘LoF’) from low-confidence (used in the ‘Missense|LC’ category). Only unique canonical transcripts for protein-coding genes were considered. LoF, synonymous and missense variants were gathered in the UKB cohort using bcftools (v.1.15.1) isec function, with the ‘-c none’ parameter to match variants by chromosome, position, reference and alternative alleles. Singleton variants were excluded from this analysis.

Identification of compound heterozygous events was performed with custom Python (v.3.7) scripts. Briefly, for each variant type (LoF, synonymous, missense) and for each gene, individuals with at least two mutations were assessed for compound heterozygosity by having at least one variant in each of the two haplotypes. In addition, for each gene, we calculated the expected number of individuals with compound heterozygosity as ${\sum }_{i=1}^{n}1-{\frac{1}{2}}^{\left(v-1\right)}$, where *v* indicates the number of variants in individual *i* in the gene. To compare the number of LoF compound heterozygous genes and events without phasing, we randomized phasing at all variants by attributing 0.5 probability for each variant to fall in either of the two haplotypes, independently for each variant.

### Essential and nonessential gene lists

We obtained lists of essential and nonessential genes from several sources (described below). For each of these gene lists, we performed Fisher’s exact tests (two-sided) for several categories of compound heterozygous genes versus noncompound heterozygous genes, considering a background of 2,150 genes with at least one individual with two LoF mutations. For synonymous and missense variants, the background included 10,119 and 14,914 genes, respectively. The following lists of genes were obtained: (1) essential in mice (*n* = 2,454) from Georgi et al.^52^ includes genes where homozygous knockout in mice results in pre-, peri- or postnatal lethality and was extracted with ortholog human gene symbols from McArthur’s laboratory^53^; (2) essential in culture (*n* = 360) core essential genes from genomic perturbation screens were obtained from Hart et al.^33^; (3) nonessential in culture (*n* = 927) putatively nonessential genes (shRNA screening) were obtained from Hart et al.^33^; (4) essential CRISPR (*n* = 684) genes essential in culture from CRISPR screening were obtained from Hart et al.^54^; (5) essential ADaM (*n* = 1,075) genes annotated by the ADaM analysis of a large collection of gene dependency profiles (CRISPR-Cas9 screens) across 855 human cancer cell lines (Project Score and Project Achilles 20Q2) were obtained from Vinceti et al.^55^; (6) essential gnomAD (*n* = 1,920) genes at the bottom LOEUF decile from gnomAD v.2.1.1 (that is, most constrained genes) were obtained from https://gnomad.broadinstitute.org/ (ref. ^4^); (7) nonessential gnomAD (*n* = 1,919) genes at the top LOEUF decile from gnomad AD v.2.1.1 (that is, least constrained genes) were obtained from https://gnomad.broadinstitute.org/ (ref. ^4^); and (8) homozygous LoF tolerant (*n* = 1,815) genes with homozygous LoF variants observed in the gnomAD cohort were obtained from Karczewski et al.^4^ (Supplementary Data 7).

### Statistics and reproducibility

This study was based on the UKB SNP array, WES and WGS datasets. Variants and samples were selected based on quality controls and ancestry as described in the SNP array, WES and WGS data processing methods. In certain analyses, only individuals including both WGS/WES and SNP array data were included. Statistical analyses, including Fisher’s exact tests, binomial and Wilcoxon tests were performed with R v.4.2. All code to reproduce analyses is publicly available.

### Reporting summary

Further information on research design is available in the Nature Portfolio Reporting Summary linked to this article.

## Online content

Any methods, additional references, Nature Portfolio reporting summaries, source data, extended data, supplementary information, acknowledgements, peer review information; details of author contributions and competing interests; and statements of data and code availability are available at 10.1038/s41588-023-01415-w.

## Supplementary information

Supplementary Information Supplementary Figs. 1–4 and Tables 1–3.

Reporting Summary

Supplementary Data 1 LoF compound heterozygous events on the UKB (WES) across human protein-coding genes. ‘num_ch_events’ is the number of individuals with compound heterozygosity for that gene; ‘num_inds_2_mut’ is the number of individuals with at least two mutations in any of the two copies of the gene; and ‘expected_ch’ is the expected number of individuals with compound heterozygous events, based their number of LoF mutations. Other columns represent presence of gene in several essential and nonessential gene lists.

## Data Availability

The lists of compound heterozygous events and genes are available in Supplementary Data 1. The phased WGS reference panel can be accessed via the UKB RAP: https://ukbiobank.dnanexus.com/landing. RAP is open to researchers who are listed as collaborators on UKB-approved access applications. Liftover was performed using a chain file provided by UCSC (https://hgdownload.cse.ucsc.edu/goldenpath/hg19/liftOver/). The publicly available subset of the Haplotype Reference Consortium dataset is available from the European Genome-Phenome Archive at the European Bioinformatics Institute, accession EGAS00001001710. Source data are provided with this paper.

## Source data

Source Data Fig. 2 Statistical source data.
Source Data Fig. 3 Statistical source data.
Source Data Extended Data Fig. 1 Statistical source data.
Source Data Extended Data Fig. 2 Statistical source data.
Source Data Extended Data Fig. 3 Statistical source data.
Source Data Extended Data Fig. 4 Statistical source data.
Source Data Extended Data Fig. 5 Statistical source data.
Source Data Extended Data Fig. 6 Statistical source data.
Source Data Extended Data Fig. 7 Statistical source data.
Source Data Extended Data Fig. 8 Statistical source data.
Source Data Extended Data Fig. 9 Statistical source data.

## Code availability

SHAPEIT5 is available under MIT license at https://github.com/odelaneau/shapeit5. This includes code to the phase_common, phase_rare, ligate and switch tools and the scripts used to phase WES and WGS data on the UKB RAP. The documentation is available at https://odelaneau.github.io/shapeit5. Code and source data to reproduce analysis and plots have been deposited in the linked Zenodo repository: 10.5281/zenodo.7828479 (ref. ^56^).
