# Supplementary material for: Accurate rare variant phasing of whole-genome and whole-exome sequencing data in the UK Biobank
Source: Nat Genet. 2023 Jun 29;55(7):1243–9. doi: 10.1038/s41588-023-01415-w (PMC10335929; doi:10.1038/s41588-023-01415-w)
Supplement: Supplementary file 1 — Supplementary Figs. 1–4 and Tables 1–3. [file 41588_2023_1415_MOESM1_ESM.pdf]

Table of content

Supplementary Figure 1: Computation of phasing probabilities.................................. 1

Supplementary Figure 2: Switch Error rates in the WGS data.................................... 2

Supplementary Figure 3: Validation of WGS phasing using trios only..........................3

Supplementary Figure 4: Validation of WGS phasing across multiple sample sizes....5

Supplementary Table 1. Summary statistics of the phased datasets........................... 6

Supplementary Table 2. Running times and cost estimates for phasing WGS data with SHAPEIT5 or Beagle5.4 on the Research Analysis Platform (RAP) of the UK Biobank.. 6

Supplementary Table 3. Running times and memory usage for phasing WGS data with SHAPEIT5 or Beagle5.4 on the Research Analysis Platform (RAP) of the UK Biobank.. 7

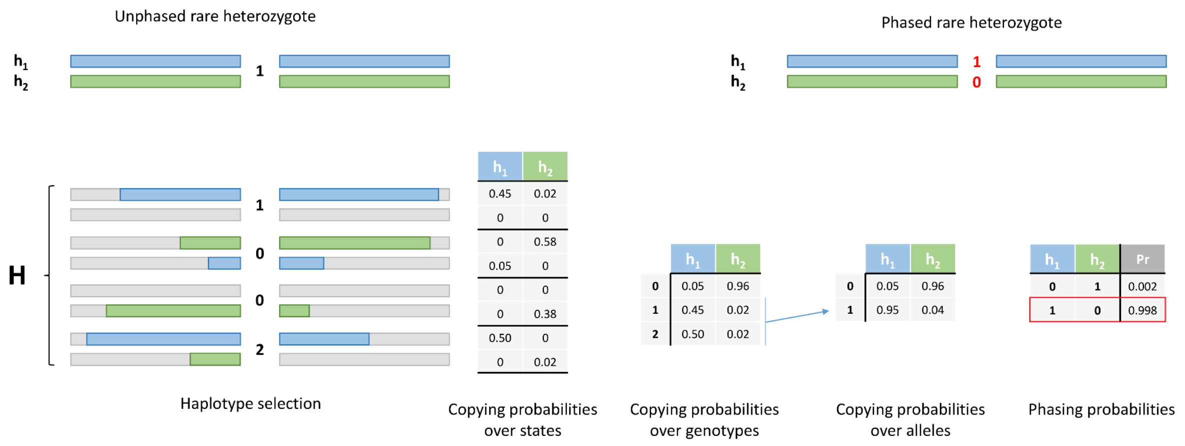

Supplementary Figure 1: Computation of phasing probabilities.
From left to right. A target sample is phased at common variants (haplotypes h_1 and h_2) and is heterozygote for a rare variant (genotype = 1). Haplotypes in the conditioning set H are chosen so that they share long matches with the target (in green and blue). At the rare variant, they are either homozygous for the major allele (genotype = 0), for the minor allele (genotype = 2) or heterozygous (genotype = 1). The haploid Li and Stephens model, as used for genotype imputation, is applied to get copying probabilities, i.e., probabilities that h_1 and h_2 copy for each haplotype in H at the rare variant. These probabilities are summed across the possible genotypes at the rare variant. Then, homozygosity is forced at heterozygous genotypes so that the probabilities can be summed per allele (0 or 1) at the rare variant, i.e. the probabilities that h_1 and h_2 carry the alleles 0 or 1 at the rare variant. Finally, these imputation probabilities are multiplied in order to get phasing probabilities: (i) h_1 = 0 and h_2 = 1 versus (ii) h_1 = 1 and h_2 = 0. The most likely phase is then assigned to get the phasing of the rare variant.

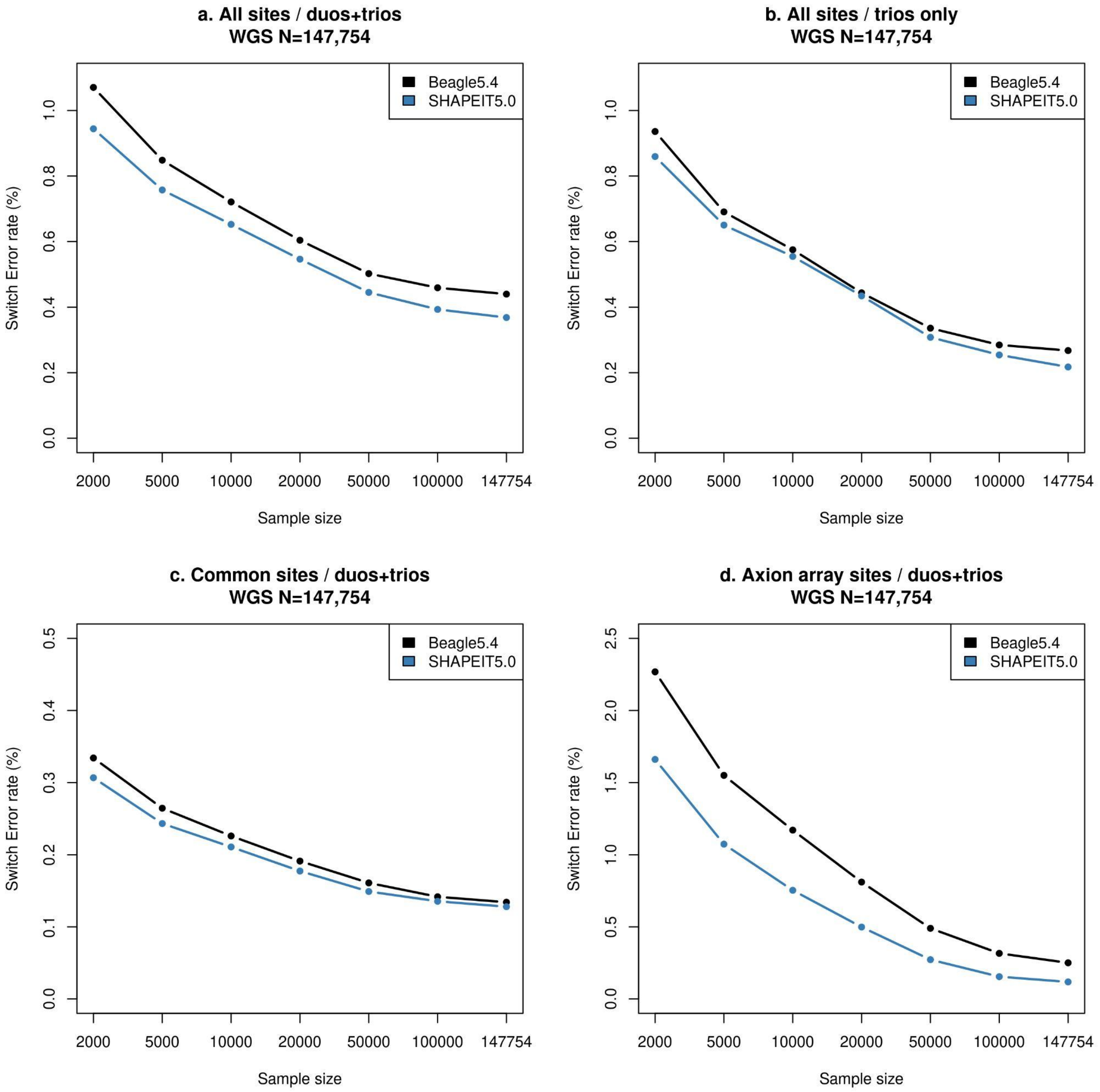

Supplementary Figure 2: Switch Error rates in the WGS data.
(**A**) SER computed at all variants in duos and trios. (**B**) SER computed at all variants just in trios. (**C**) SER computed in duos and trios for common variants (MAF ≥ 0.1%). (**D**) SER computed in duos and trios for variants included on the Axiom array. SER was computed for multiple downsampling experiments comprising 2000, 5000, 10000, 20000, 50000, 100000 and 147754 samples (x-axis). SHAPEIT5 and Beagle5.4 are shown in blue and black, respectively.

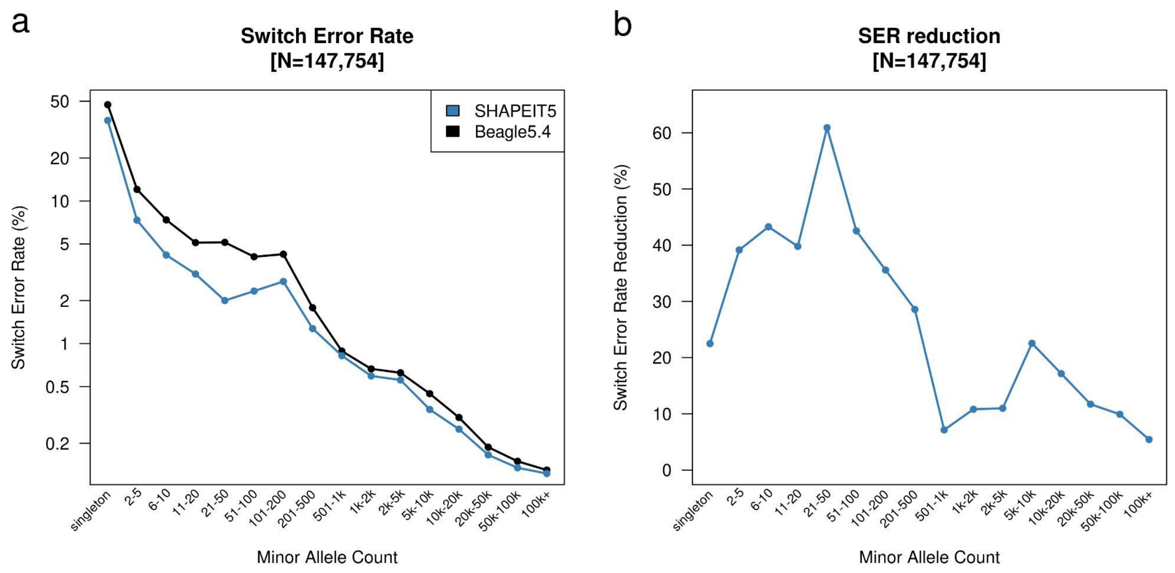

Supplementary Figure 3: Validation of WGS phasing using trios only.
(**A**) SER stratified by minor allele count when using only trios as validation (instead of duos and trios). (**B**) Corresponding SER reductions. Results for SHAPEIT5 and Beagle5.4 are shown in blue and black, respectively.

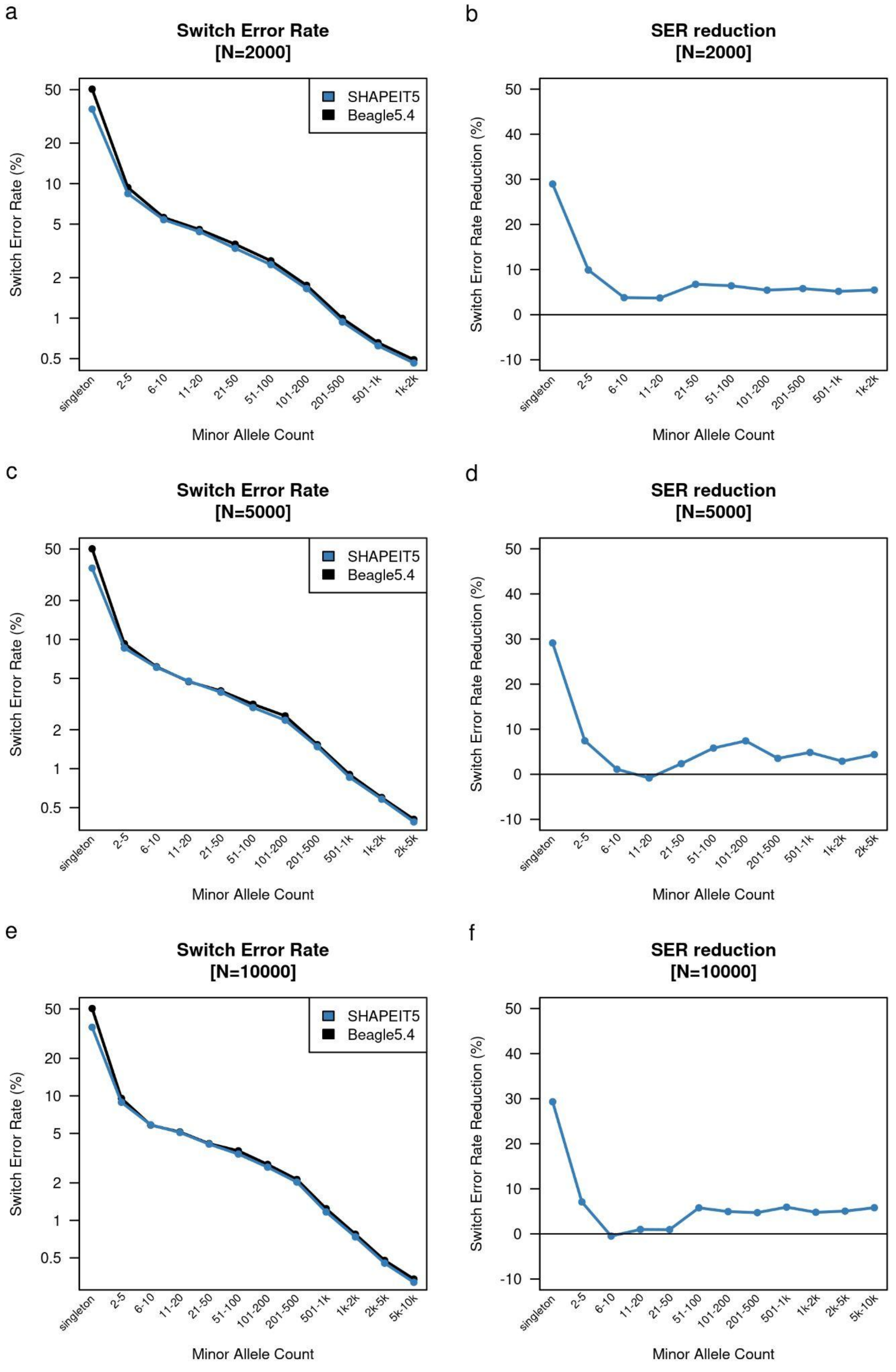
a
Switch Error Rate
[N=2000]
SHAPEIT5
Beagle5.4
Switch Error Rate (%)
50
20
10
5
2
1
0.5
singleton
2-5
6-10
11-20
21-50
51-100
101-200
201-500
501-1k
1k-2k
Minor Allele Count
b
SER reduction
[N=2000]
Switch Error Rate Reduction (%)
50
40
30
20
10
0
-10
Minor Allele Count
c
Switch Error Rate
[N=5000]
2k-5k
d
SER reduction
[N=5000]
e
Switch Error Rate
[N=10000]
5k-10k
f
SER reduction
[N=10000]

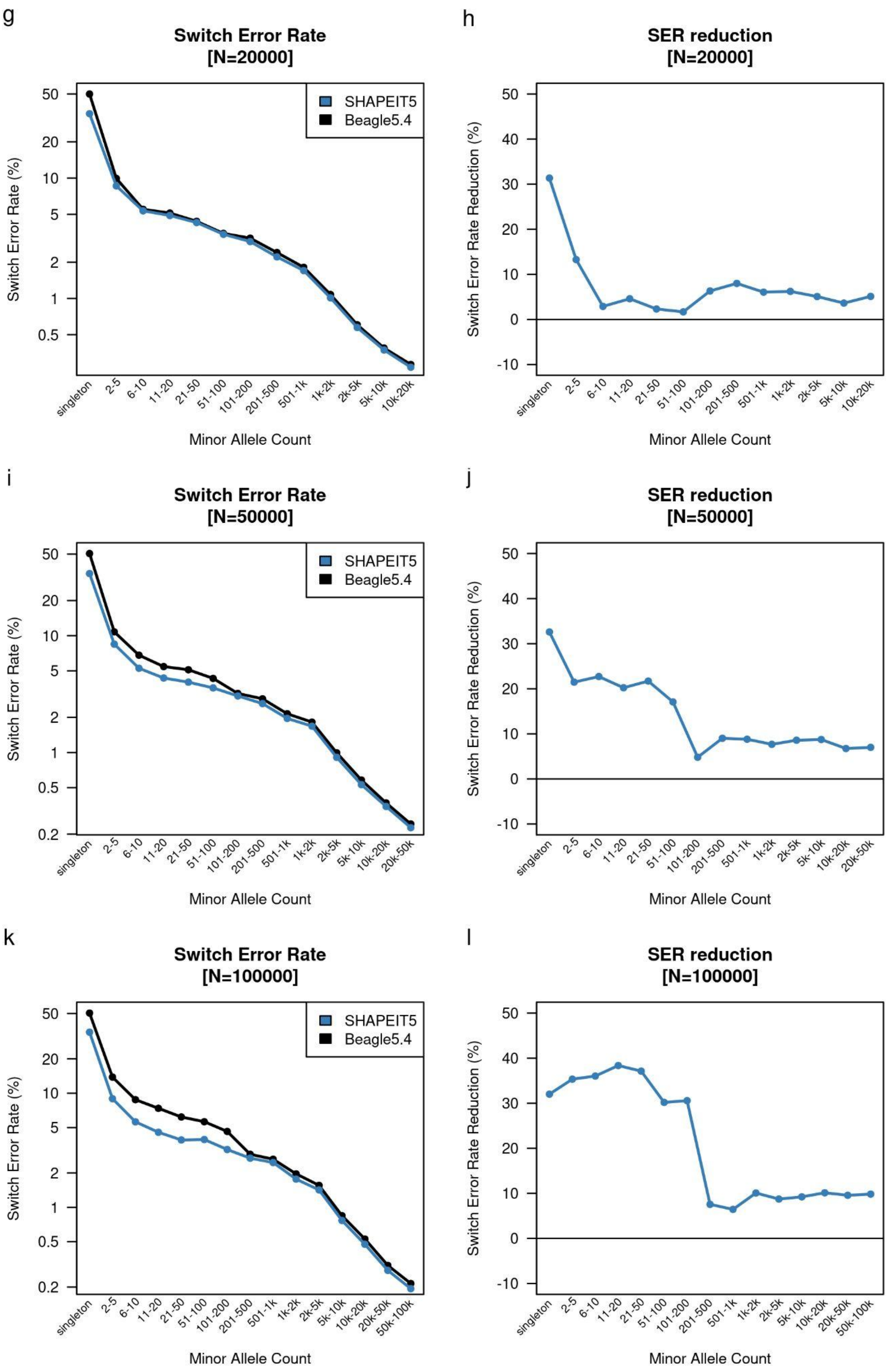

Supplementary Figure 4: Validation of WGS phasing across multiple sample sizes. Plots on the left show the SER stratified by minor allele count when using trios and duos as validation for, top to bottom, 2000, 5000, 10000, 20000, 50000, 100000 samples. Results for SHAPEIT5 and Beagle5.4 are shown in blue and black, respectively. Plots on the right show the corresponding SER reductions.

Supplementary Table 1. Summary statistics of the phased datasets.

	SNP array	WES+array	WGS	WGS, chr20
Number of variants	670,741	26,199,614	603,925,301	13,780,190
Number of rare < 0.1%MAF		25,222,097	583,262,899	13,304,181
Number of common ≥ 0.1%MAF		977,517	20,662,402	476,009
Number of samples	486,442	452,644	150,119	147,754
Number of white British trios	897	719	31	31
Number of white British duos	4373	3104	432	432

Supplementary Table 2. Running times and cost estimates for phasing WGS data with SHAPEIT5 or Beagle5.4 on the Research Analysis Platform (RAP) of the UK Biobank.

Software	Step	Total time	Time per job	Virtual machine	Cost on-demand	Cost on-spot
Beagle5.4	Phasing all variants	24:45	NA	mem3_ssd1_v2_x64	56.86	11.37
Beagle5.4	Indexing	04:10	NA	mem3_ssd1_v2_x8	0.96	0.19
Beagle5.4	Total chr20	28:55	NA	NA	57.82	11.56
Beagle5.4	Total whole genome	60d, 5:50	NA	NA	2890	578
SHAPEIT5	Phasing common variants	38:52	02:26	mem3_ssd1_v2_x32	45.4	8.21
SHAPEIT5	Ligation	01:24	01:24	mem3_ssd1_v2_x2	0.10	0.02
SHAPEIT5	Phasing rare variants	16:33	01:02	mem3_ssd1_v2_x32	19.33	3.87
SHAPEIT5	Concatenating + Indexing	04:28	04:28	mem3_ssd1_v2_x2	0.33	0.07
SHAPEIT5	Total chr20	61:17	09:20	NA	65.16	13.03
SHAPEIT5	Total whole genome	127d, 16:20	NA	NA	3258	651.6

Supplementary Table 3. Running times and memory usage for phasing WGS data with SHAPEIT5 or Beagle5.4 on the Research Analysis Platform (RAP) of the UK Biobank.

Software	Step	Virtual Machine	User time (s)	Elapsed time* (hh:mm:ss)	Memory Usage (Gb)
SHAPEIT5	Phasing common variants	mem3_ssd1_v2_x32	3026478	38:52	9.31 (149)**
SHAPEIT5	Phasing rare variants	mem3_ssd1_v2_x32	681317	16:33	22.7 (364)**
Beagle5.4	Phasing all variants	mem3_ssd1_v2_x64	4647342	24:45	501***

(*) Elapsed time on the corresponding Virtual machine
(**) Average memory usage of SHAPEIT5 per 4Mb chunk of data and sum over all chunks is given in brackets.
(***) Memory usage of Beagle5.4 is given for the whole chromosome 20 when run with *java --Xmx460G*.
