# Supplementary material for: Accurate rare variant phasing of whole-genome and whole-exome sequencing data in the UK Biobank
Source: Nat Genet. 2023 Jun 29;55(7):1243–9. doi: 10.1038/s41588-023-01415-w (PMC10335929; doi:10.1038/s41588-023-01415-w)
Supplement: Supplementary file 2 — Reporting Summary [file 41588_2023_1415_MOESM2_ESM.pdf]

nature portfolio

Corresponding author(s): Olivier Delaneau

Last updated by author(s): Apr 14, 2023

Reporting Summary

Nature Portfolio wishes to improve the reproducibility of the work that we publish. This form provides structure for consistency and transparency in reporting. For further information on Nature Portfolio policies, see our Editorial Policies and the Editorial Policy Checklist.

Statistics

For all statistical analyses, confirm that the following items are present in the figure legend, table legend, main text, or Methods section.

n/a	Confirmed	
☐	☒	The exact sample size (*n*) for each experimental group/condition, given as a discrete number and unit of measurement
☒	☐	A statement on whether measurements were taken from distinct samples or whether the same sample was measured repeatedly
☐	☒	The statistical test(s) used AND whether they are one- or two-sided<br>*Only common tests should be described solely by name; describe more complex techniques in the Methods section.*
☒	☐	A description of all covariates tested
☒	☐	A description of any assumptions or corrections, such as tests of normality and adjustment for multiple comparisons
☐	☒	A full description of the statistical parameters including central tendency (e.g. means) or other basic estimates (e.g. regression coefficient) AND variation (e.g. standard deviation) or associated estimates of uncertainty (e.g. confidence intervals)
☐	☒	For null hypothesis testing, the test statistic (e.g. *F*, *t*, *r*) with confidence intervals, effect sizes, degrees of freedom and *P* value noted<br>*Give P values as exact values whenever suitable.*
☐	☒	For Bayesian analysis, information on the choice of priors and Markov chain Monte Carlo settings
☒	☐	For hierarchical and complex designs, identification of the appropriate level for tests and full reporting of outcomes
☐	☒	Estimates of effect sizes (e.g. Cohen's *d*, Pearson's *r*), indicating how they were calculated

Our web collection on statistics for biologists contains articles on many of the points above.

Software and code

Policy information about availability of computer code

Data collection

No software was used for the data collection of this study.

Data analysis

Third party code and programs used in this study are all publicly available:

Samtools v1.16 (based on htslib v1.16)
BCFtools v1.16 (based on htslib v1.16)
GLIMPSE v1.1.1
PLINK v2.0
R v4.2.2
Beagle v5.4
Python v3.7
PICARD v3.0.0

Custom code and programs developed as part of the study:

SHAPEIT v5.0.0-beta (https://doi.org/10.5281/zenodo.7828479)
Python3 scripts (https://doi.org/10.5281/zenodo.7828479)
R scripts (https://doi.org/10.5281/zenodo.7828479)

For manuscripts utilizing custom algorithms or software that are central to the research but not yet described in published literature, software must be made available to editors and reviewers. We strongly encourage code deposition in a community repository (e.g. GitHub). See the Nature Portfolio guidelines for submitting code & software for further information.

Data

Policy information about availability of data

All manuscripts must include a data availability statement. This statement should provide the following information, where applicable:

- Accession codes, unique identifiers, or web links for publicly available datasets
- A description of any restrictions on data availability
- For clinical datasets or third party data, please ensure that the statement adheres to our policy

The publicly available subset of the Haplotype Reference Consortium dataset is available from the European Genome-Phenome Archive at the European Bioinformatics Institute, accession EGAS00001001710.

The UK Biobank data was accessed under the project 66995.

The lists of compound heterozygous events and genes are available in Supplementary Data 1.

The phased WGS reference panel can be accessed via the UKB research analysis platform (RAP): https://ukbiobank.dnanexus.com/landing.

Liftover was performed using a chain file provided by UCSC (https://hgdownload.cse.ucsc.edu/goldenpath/hg19/liftOver/).

Human research participants

Policy information about studies involving human research participants and Sex and Gender in Research.

Reporting on sex and gender	No sex- or gender-based analyses were conducted, as we only report phasing and imputation results for the autosomes.
Population characteristics	We identify populations using self reported which is confirmed by Principal Component Analysis (provided by the UK Biobank)
Recruitment	N/A (Performed by the UK Biobank)
Ethics oversight	N/A (Performed by the UK Biobank)

Note that full information on the approval of the study protocol must also be provided in the manuscript.

Field-specific reporting

Please select the one below that is the best fit for your research. If you are not sure, read the appropriate sections before making your selection.

☒ Life sciences ☐ Behavioural & social sciences ☐ Ecological, evolutionary & environmental sciences

For a reference copy of the document with all sections, see nature.com/documents/nr-reporting-summary-flat.pdf

Life sciences study design

All studies must disclose on these points even when the disclosure is negative.

Sample size	Sample sizes are clearly indicated in the manuscript for each of the different data sets. Sample sizes were determined as the total number of available individuals in each call sets. When assessing phasing accuracy, sample size were determined as the total number of available individuals, excluding parental genomes. Parent-offspring pairs were determined using the kinship and IBS0 estimates provided by the UK Biobank research team.
Data exclusions	No data was excluded.
Replication	We emphasize the importance of reproducibility in our research and have taken the necessary steps to ensure it. All of the code and scripts used in our experiments are available in an open-source format and can be accessed through our GitHub repository. We have also documented the versions of the software and tools used to generate the results, enabling other researchers to replicate our experiments with ease.
Randomization	Randomization was not used since there are no experimental groups.
Blinding	Blinding is not relevant to this study since no group allocation occurs.

Reporting for specific materials, systems and methods

We require information from authors about some types of materials, experimental systems and methods used in many studies. Here, indicate whether each material, system or method listed is relevant to your study. If you are not sure if a list item applies to your research, read the appropriate section before selecting a response.

Materials & experimental systems

n/a	Involved in the study
☒	☐ Antibodies
☒	☐ Eukaryotic cell lines
☒	☐ Palaeontology and archaeology
☒	☐ Animals and other organisms
☒	☐ Clinical data
☒	☐ Dual use research of concern

Methods

n/a	Involved in the study
☒	☐ ChIP-seq
☒	☐ Flow cytometry
☒	☐ MRI-based neuroimaging
